# Anti-Apoptotic Effects of Carotenoids in Neurodegeneration

**DOI:** 10.3390/molecules25153453

**Published:** 2020-07-29

**Authors:** Han-A Park, Mary Margaret Hayden, Sydni Bannerman, Joseph Jansen, Kristi M. Crowe-White

**Affiliations:** Department of Human Nutrition and Hospitality Management, College of Human Environmental Sciences, The University of Alabama, Tuscaloosa, AL 35487, USA; mmhayden@crimson.ua.edu (M.M.H.); stbannerman@crimson.ua.edu (S.B.); jpjansen@crimson.ua.edu (J.J.); kcrowe@ches.ua.edu (K.M.C.-W.)

**Keywords:** apoptosis, mitochondria, carotenoid, neurodegeneration, antioxidant

## Abstract

Apoptosis, programmed cell death type I, is a critical part of neurodegeneration in cerebral ischemia, Parkinson’s, and Alzheimer’s disease. Apoptosis begins with activation of pro-death proteins Bax and Bak, release of cytochrome c and activation of caspases, loss of membrane integrity of intracellular organelles, and ultimately cell death. Approaches that block apoptotic pathways may prevent or delay neurodegenerative processes. Carotenoids are a group of pigments found in fruits, vegetables, and seaweeds that possess antioxidant properties. Over the last several decades, an increasing number of studies have demonstrated a protective role of carotenoids in neurodegenerative disease. In this review, we describe functions of commonly consumed carotenoids including lycopene, β-carotene, lutein, astaxanthin, and fucoxanthin and their roles in neurodegenerative disease models. We also discuss the underlying cellular mechanisms of carotenoid-mediated neuroprotection, including their antioxidant properties, role as signaling molecules, and as gene regulators that alleviate apoptosis-associated brain cell death.

## 1. Introduction

**Figure 1:**
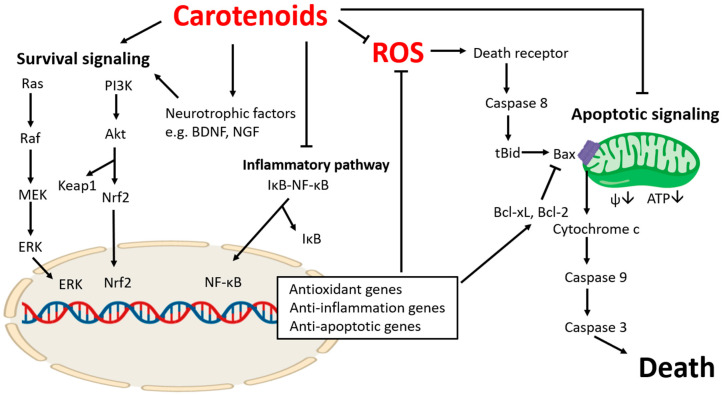
Protective roles of carotenoids during neuronal apoptosis. ROS: reactive oxygen species. ROS can trigger both death receptor- and mitochondria-mediated apoptotic pathways. As an antioxidant, carotenoids scavenge ROS to prevent ROS-induced apoptotic pathways. Carotenoids manipulate numerous cell-signaling pathways including Erk, Akt, and NF-κB to enhance transcription of genes that promote antioxidant defense, anti-inflammation, and anti-apoptosis, illustration done using BioRender.

Apoptosis, programmed cell death type I, naturally occurs during development and maturation of the healthy brain [1,2]. However, apoptotic pathways also play an important role in brain-associated diseases that are caused by loss of functional populations of neurons. During neurotoxic stimulation, such as oxidative stress, excitotoxicity, or neuroinflammation, brain cells undergo death receptor-mediated (extrinsic) apoptosis, mitochondria-mediated (intrinsic) apoptosis, or a combination of both. Activated death receptors such as tumor necrosis factor receptor 1 (TNFR1) form a trimer, recruiting adapter proteins such as TNFR1-associated death domain protein (TRADD), TNRF-associated factor (TRAF) 2, cellular inhibitor of apoptosis (cIAP) 1 and 2, and Fas-associated death domain protein (FADD). FADD further recruits procaspase 8 and converts it to its active form. Death receptor-mediated caspase 8 activation results in cleavage of Bcl-2 interacting protein (Bid), a pro-apoptotic BH3-only protein, producing truncated Bid (tBid) [3]. tBid is translocated to the mitochondrial membrane and recruits multidomain proapoptotic Bcl-2 proteins such as Bcl-2-associated X protein (Bax). Bax is oligomerized with itself or other pro-apoptotic proteins such as Bak, making the mitochondrial membrane permeable (Figure 1).

Unlike BH3-only and multidomain pro-apoptotic proteins, B-cell lymphoma-2 (Bcl-2), and B-cell lymphoma-extra large (Bcl-xL) contain a BH4 domain, a key domain with anti-apoptotic activities. There, anti-apoptotic Bcl-2 family proteins bind directly to pro-apoptotic Bcl-2 proteins preventing oligomerization. However, excess activation of pro-apoptotic Bcl-2 proteins causes loss of mitochondrial membrane integrity leading to the release of cytochrome c. In healthy mitochondria, cytochrome c is found on the inner membrane and acts as an electron carrier in the electron transport chain. However, during mitochondrial outer membrane permeabilization, cytochrome c is released into the cytoplasm due to its hydrophilicity. Cytoplasmic cytochrome c interacts with apoptotic protease activating factor (Apaf) 1 and dATP to form an apoptosome. The apoptosome activates caspase 9, which further activates executor caspases such as caspase 3 that degrade functional and structural proteins in the cell. This ultimately leads to neuronal death. Although opening of the mitochondrial permeability transition pore (mtPTP), a large mitochondrial inner membrane death channel [4,5,6], occurs in both apoptotic and necrotic neurons, it may be regulated by Bcl-2 family proteins. Bcl-xL binds directly to the c-subunit of the F_1_Fo ATP synthase [7], a voltage-sensitive channel that acts like a mtPTP [8,9], preventing proton leak through the pore. Bcl-xL is reported to form a complex with other mtPTP candidates such as the voltage-dependent anion channel and adenine nucleotide translocator to prevent mitochondrial dysfunction [10,11].

Enhanced neuronal apoptosis after cerebral ischemia is well documented in both rodent models of stroke (focal cerebral ischemia) and cardiac arrest (global cerebral ischemia) [12,13,14,15,16,17]. In vitro models that mimic cerebral ischemia including treatment with glutamate, hydrogen peroxide, hypoxia, and oxygen-glucose deprivation consistently show increased levels of apoptosis [18,19,20,21]. Although there are numerous studies demonstrating involvement of apoptotic pathways during ischemic insult, key findings indicate that cerebral ischemia (1) alters the proportion of pro-apoptotic vs. anti-apoptotic Bcl-2 proteins, (2) increases mitochondrial membrane permeability, and (3) activates caspases in both mitochondria-dependent and independent manners.

Apoptosis also plays an important role in Alzheimer’s disease [22,23,24]. Accumulation of amyloid-β induces both death receptor-mediated extrinsic and mitochondria-mediated intrinsic apoptosis. For example, TNFR1 is necessary during amyloid-β-induced neuronal death [25], and amyloid-β increases both mRNA and protein levels of Fas ligand [26]. In addition, treatment with amyloid-β manipulates expression of Bcl-2 family proteins and induces cytochrome c release from mitochondria [27,28,29,30]. Amyloid-β-mediated activation of initiator caspases including caspase 8 [31,32] and executor caspases like caspase 3 [33] has been widely investigated. Caspases carry out proteolytic cleavage of amyloid precursor protein (APP) producing amyloid-β [34,35,36]. Aggregation of tau, the neurofibrillary tangles found in both Alzheimer’s and Parkinson’s-affected brains, is regulated by caspases. The Asp421 residue on the C-terminal of tau is subjected to caspases, and increased proteolytic cleavage of tau is found in apoptotic neurons [37,38]. Therefore, apoptosis is involved in both initiation and propagation of the pathology of Alzheimer’s disease.

Loss of dopaminergic neurons in the substantia nigra results in Parkinson’s disease. Although autophagy and necrosis are also involved during neurodegeneration, apoptotic features including increased abundance of pro-apoptotic Bcl-2 protein, activation of caspases, and cytochrome c release are found during progression of Parkinson’s disease [39,40,41]. Mitochondrial dysfunction, particularly impairment of complex I, is a key feature of Parkinson’s pathology [42,43,44]. Currently, application of complex I inhibitors like 1-methyl-4-phenyl-1,2,3,6-tetrahydropyridine (MPTP) and rotenone are widely used to mimic Parkinsonian pathology in research [45,46]. Additionally, mutations of PARK genes or abnormality of PARK gene products is highly associated with mitochondria-mediated neuronal damage. Oligomerization of α-synuclein, encoded by the PARK1 gene, triggers apoptosis [47]. Depletion of Parkin or PINK1, encoded by the PARK2 and PARK6 genes, respectively, impairs removal of dysfunctional mitochondria and increases cellular reactive oxygen species (ROS) load [48]. DJ-1, encoded by the PARK7 gene, binds directly to the F_1_Fo ATP synthase and anti-apoptotic Bcl-xL to improve mitochondrial energy metabolism and dopaminergic neuron survival [49].

Application of pharmacological inhibitors and genetic modifications that block oligomerization of pro-apoptotic Bcl-2 proteins [50,51] or enhance the function of Bcl-2 or Bcl-xL protect neurons from injury [12,18,52,53]. Treatment with caspase inhibitors has protective effects in cerebral ischemia and neurodegeneration models [18,54,55,56]. However, there are significant barriers to applying molecular-based strategies to treat brain-associated diseases in humans. In recent decades, an increasing number of researchers have highlighted the potential significance of nutrients and dietary phytochemicals as neuroprotectants [57,58,59,60]. Since neurodegeneration occurs gradually over a number of years, improving life-style factors like diet may delay apoptotic neuronal loss.

## 2. Overview of Carotenoids

Carotenoids are a family of yellow, orange, and red pigments found in plants, fungi, bacteria, and algae. Carotenoids play an important role in photosynthesis by extending the range of wavelengths of light that drive the reaction forward. In addition, carotenoids protect photosynthetic organisms from damage caused by excessive exposure to light [61,62]. There are over 600 types of carotenoids found in nature that provide a large range of health benefits. Carotenoids can be categorized into six different groups according to their chemical structures: hydrocarbons, hydroxycarotenoids, epoxycarotenoids, aldehydes, ketones, and carboxylic acids [63]. The many types of carotenoids are categorized into two large groups, the carotenes and the xanthophylls based on their chemical components. Carotenes consist of lycopene, β-carotene, and α-carotene based on their structure containing only a parent hydrocarbon chain [64]. Xanthophylls consist of structures that contain oxygen as a functional group such as beta-cryptoxanthin, neoxanthin, astaxanthin, canthaxanthin, zeaxanthin, fucoxanthin, and lutein [64].

A large number of carotenoids are present in the human diet, with foods such as fruits and vegetables containing rich pigmentation. It has also been noted that nearly 20 types of carotenoids are present in the blood and tissues of the human body [65]. Experimentation with supplementation of carotenoids can affect cellular redox status, gene expression, immune responses, cell growth, and development [66]. Studies are continuing to be conducted to fully understand the extensive effects carotenoids have on the brain and brain-related diseases.

### 2.1. Lycopene

Lycopene is a carotenoid responsible for the red-pink color of certain vegetables and fruits, including tomatoes, grapefruit, watermelon, and guava. Chemically, it is a hydrocarbon carotenoid, composed strictly of carbon and hydrogen assembled from eight isoprene units. In the fresh fruit and vegetable form, lycopene exists predominantly as trans-lycopene. Upon exposure to heat, trans-lycopene undergoes isomerization to cis-lycopene [67]. Numerous clinical studies have demonstrated the greater absorption and bioavailability of cis-versus trans-lycopene [68,69,70]. Bioavailability remains stable throughout heat treatment [71,72], allowing lycopene to retain its biological function during food processing. Extensive research has been published on lycopene, and it has potential benefits in regard to cancer, cardiovascular disease, and inflammatory disease [73]. The efficiency of lycopene crossing the blood–brain barrier remains controversial. Oral administration of lycopene (10, 30, and 50 mg/kg) increases lycopene levels in liver, the adrenals, spleen, lymph nodes, and intestinal tissues in dogs, but lycopene was not detected in the brain [74]. However, oral administration of lycopene (8 mg/kg) increased the lycopene concentration in mouse brain, and improvement of this delivery method by using a lycopene-loaded microemulsion significantly enhanced brain distribution of lycopene [75]. Lycopene acts as an antioxidant with greater singlet oxygen quenching abilities than other carotenoids such as β-carotene, lutein, and zeaxanthin [76]. Application of lycopene has been shown to protect brain cells from oxidative stress-induced damage. Treatment with lycopene inhibits both lipid peroxidation of membranes and accumulation of hydrogen peroxide and superoxide in both in vitro and in vivo neurodegeneration models [77,78,79,80,81]. Interestingly, pro-oxidant effects of lycopene have been reported under co-treatment with a lipid-soluble radical generator in fibroblasts [82], thus, the efficacy of lycopene as an antioxidant may differ by both oxidant and cell type. Additionally, lycopene upregulates intracellular antioxidant defense system components including superoxide dismutase, catalase, glutathione peroxidase, and glutathione [79,81,83]. The antioxidant properties of lycopene are particularly important in protecting mitochondria [77,80,83]. Mitochondria are the central oxygen consuming organelle by virtue of housing the electron transport chain, but mitochondria are also responsible for production of ROS [84]. Excess ROS accumulation and impaired antioxidant defenses result in mitochondrial dysfunction, and mitochondrial dysfunction results in depletion of energy, initiation of apoptosis, and ultimately neuronal death in the brain [85,86]. Treatment with lycopene prevents loss of mitochondrial inner membrane potential during ROS challenge [78,83,87]. Qu et al. show that lycopene improves energy metabolism in primary cortical neurons by preventing loss of complex I, II, III, and IV activity during amyloid-β treatment [77]. This same group also suggests that lycopene is capable of protecting mitochondrial DNA that encodes NADH dehydrogenase subunit 6 and cytochrome c oxidase subunit I, key subunits found in complexes I and IV, respectively [77]. Although not yet confirmed, lycopene-mediated energy retention may occur via protection of other mitochondrial DNA like the ATP6 and ATP8 genes, which encode the a-subunit of F_1_Fo ATP synthase (Complex V), the enzyme complex responsible for ATP production [88].

Anti-apoptotic functions of lycopene in neurodegeneration have been reported relatively recently. Lycopene prevents loss of anti-apoptotic proteins including Bcl-2 and Bcl-xL [80,89], while inhibiting pro-apoptotic proteins like Bax [78,80] under neurotoxic conditions. Lycopene protects primary hippocampal neurons from apoptotic death by blocking opening of mtPTP [90]. Neurons treated with lycopene have decreased mitochondrial depolarization and cytochrome c release, indicating retention of mitochondrial membrane integrity [77,83]. Pathological concentrations of intracellular calcium trigger opening of mtPTP [91]. Zhang et al. showed that treatment with lycopene increases activity of the Ca^2+^-ATPase and Ca^2+^-Mg^2+^-ATPase, transport proteins that assist with clearance of calcium from the cell [79]. Thus, lycopene may prevent mtPTP-associated neuronal death by regulating calcium homeostasis. In addition, lycopene prevents activation of caspase 3 [78]. Although further investigation is required to elucidate the exact molecular mechanism behind lycopene-mediated inhibition of apoptosis, lycopene may also regulate the transcription of genes that control apoptosis [78,79].

Treatment with lycopene increases phosphorylation of Akt [78,79]. Akt, also called protein kinase B, is phosphorylated upon activation of phosphoinositide 3-kinase (PI3K), and phosphorylated Akt regulates transcription factors (Figure 1) such as nuclear factor erythroid 2-related factor 2 (Nrf2) and NF-κB, influencing gene expression. Indeed, lycopene is shown to manipulate Nrf2 and NF-κB targets, alleviating oxidative stress or inflammation-associated damage in brain cells [80,92]. Lycopene mediates the functionality of the cyclooxygenase-2 (COX-2) pathway, which is primarily responsible for production of pro-inflammatory prostaglandins [93,94], and lycopene reduces the release of inflammatory cytokines and chemokines including interleukin (IL)-6 and IL-8 and acute phase proteins such as C-reactive protein (CRP) [95,96]. Lycopene has been shown to attenuate cognitive deficits by reducing inflammation in the gut–liver–brain axis as well as improving glycolipid metabolism [97]. Recent studies suggest that anti-inflammatory effects of lycopene may help alleviate neuropsychiatric diseases such as post-traumatic stress disorder and depression [98,99]. Treatment with lycopene improves stress-associated behavior in rodents by preventing accumulation of inflammatory cytokines in the brain [98,99]. Additionally, Akt was previously shown to upregulate expression of the Bcl-2 gene by activating the cAMP-response element (CRE) [100], and Nrf2 [101] bind directly to the Bcl-x gene, contributing to the retention of protective Bcl-2 and Bcl-xL. Lycopene also activates the mitogen-activated protein kinase (MAPK)/extracellular signal-regulated kinase (Erk) pathway in the brain [102]. The MAPK/Erk pathway (Figure 1) includes a series of kinase activities including a serine/threonine-specific protein kinase Raf, mitogen-activated protein kinase kinase (MEK/MAPKK), and Erk. Phosphorylated Erk translocates into the nucleus and activates transcription factors such as Elk-1 and Msk regulating synaptic plasticity [103,104]. 

Lycopene treatment in vitro and in vivo using intracerebroventricular administration show improved production of neurotrophic factors including brain-derived neurotrophic factor (BDNF) and nerve growth factor (NGF) during neurotoxic challenge [78,105]. BDNF blocks neuronal apoptosis during excitotoxicity by inducing phosphorylation of Akt at Ser473 and Thr308 sites [106,107]. Akt is shown to upregulate genes containing CRE such as Bcl-2 [100,106]. NGF also plays a role in regulating apoptosis via activation of PI3K/Akt and MAPK/Erk pathways [2,108]. Interaction between NGF and tropomyosin receptor kinase A (TrkA) activates Erk signaling to phosphorylate the BH3-only protein Bim, and this inactivates the pro-apoptotic function of Bim [109,110].

### 2.2. β-Carotene

β-carotene is a carotenoid exhibiting the red and orange pigments in certain fruits and vegetables such as carrots, sweet potatoes, and squash. It contains an unsaturated hydrocarbon chain with β-rings at both ends. Symmetric cleavage of β-carotene by β-carotene-15,15-oxygenase releases two molecules of retinal, an aldehyde form of vitamin A [111,112,113]. Retinal is further metabolized to retinoic acid or to retinol and retinyl ester, an acid, alcohol, and ester form of vitamin A, respectively [111,112]. Therefore, β-carotene is an important provitamin A. Nuclear receptors including retinoic acid receptor (RAR) and retinoid X receptor (RXR) are dimerized and regulate genes containing retinoid acid response elements. Studies suggest that RAR and RXR may be required during neurogenesis and for neuroprotection against apoptosis [114,115].

β-carotene decreases accumulation of ROS including hydrogen peroxide and lipid peroxide radicals [116,117]. β-carotene is characterized as a direct acting low molecular weight antioxidant due to its scavenger nature. The ability of β-carotene to work against lipid peroxidation and decrease oxidative stress makes it an important factor in brain-related diseases. [118]. Lower levels of β-carotene in the cerebral cortex are associated with traumatic brain injury [119]. Unlike vitamin A, which can produce oxidative stress with excess treatment, β-carotene does not show pro-oxidant activity in the brain, and it has been thus suggested as a safer antioxidant [116]. Treatment with β-carotene improves retention of intracellular antioxidants such as glutathione and superoxide dismutase during neurotoxicity [118,120]. β-carotene increases protein levels of nuclear Nrf2 and upregulates downstream targets containing antioxidant response elements (ARE) such as NAD(P)H quinone dehydrogenase and heme oxygenase-1 [120]. Oxidative stress is a major contributor to neuronal apoptosis during neurotoxicity, thus, treatment with β-carotene may prevent neuronal loss in ROS-associated brain diseases. Indeed, β-carotene is shown to interfere with apoptotic pathways in the brain. Treatment with β-carotene prevents loss of Bcl-2 and accumulation of Bax and caspase 3 in a mouse model of traumatic brain injury [120].

In addition to its protection against oxidative stress, β-carotene may play a beneficial role during neuronal development. β-carotene improves cognitive and social behavior of newborn mice [121]. Oral supplementation of β-carotene increases levels of BDNF in mice [121,122]. Interaction between N-terminal cleaved mature BDNF (129–247 amino acid residues) and a receptor like tropomyosin receptor kinase B (TrkB) activates PI3K/Akt pathways, and this interaction activates cAMP response element-binding protein (CREB), a transcription factor that regulates genes that control neuronal development and plasticity [123,124]. N-terminal (19–128 amino acids) containing proBDNF binds to the P75 neurotrophin receptor, a type of tumor necrosis factor (TNF) receptor that mediates apoptosis, indicating that β-carotene-mediated neuronal development and survival may be via regulation of mature BDNF/proBDNF in the brain.

### 2.3. Astaxanthin

Astaxanthin is a red-orange carotenoid found naturally in algae, yeast, salmon, trout, krill, shrimp, and crayfish [125]. It is a xanthophyll carotenoid made up of carbon, hydrogen, and oxygen [126]. Chemically, it consists of two terminal rings joined by a polyene chain, with a hydroxyl group and a carbonyl group located on each terminal ring [126]. The polyene chain consists of conjugated double bonds that result in delocalized electrons that can be readily donated and thus provide astaxanthin with strong antioxidant capabilities [127]. Studies have shown astaxanthin as possessing anti-lipid peroxidation [128], anti-inflammatory [129], anti-diabetic [130], and anti-cancer activity [131], as well as cardiovascular disease prevention [132] and immuno-modulation [133]. Astaxanthin does have a low oral bioavailability, but absorption is enhanced by combined consumption with fats [134]. Its lipophilic nature allows it to cross the blood–brain barrier, and all-trans-astaxanthin is detected in rat hippocampus and cortex after oral administration of astaxanthin (100 mg/kg) [135]. Astaxanthin has been shown to exert anti-oxidative protective effects in neurological disorders and is shown to decrease markers of lipid oxidation including malodialdehyde, decrease protein levels of ROS-generating enzymes such as NADPH oxidase 4 and p22phox protein, and increase antioxidant enzymes such as superoxide dismutase [136]. Astaxanthin was also shown to increase protein levels of Nrf2 and p62, thereby protecting against oxidative stress [136,137]. Nrf2 is normally sequestered in the cytosol bound to Keap1 [138]. Oxidative stress and an increase in p62, which competes to bind to Keap1 [139], cause Nrf2 to translocate to the nucleus to activate ARE [138]. Astaxanthin also protects against apoptosis by regulating mitochondrial proteins [136,137,140,141]. Treatment with astaxanthin increased levels of Bcl-2 [136,140,141] and decreased levels of Bax and cleaved caspase 3 [136,137,140,141]. By increasing levels of Bcl-2, astaxanthin prevents the release of cytochrome c into the cytosol and thereby prevents apoptosis [142]. Activated Akt has been shown to phosphorylate Bad, and this prevents the activation of caspase 9 and caspase 3 to protect mitochondria [143]. Treatment with astaxanthin prevents loss of mitochondrial membrane potential in rat hippocampal neurons [140].

Astaxanthin also appears to exert protective effects via anti-inflammatory pathways [136,137,141]. The NF-κB pathway is a pro-inflammatory pathway that regulates the production of cytokines and chemokines [144]. While in an inactive state, NF-κB binds to inhibitor of nuclear factor kappa B (IκBα) and is sequestrated in the cytoplasm. However, while in an active state, IκBα is dissociated from NF-κB, and NF-κB is translocated to the nucleus, regulating gene expression. Treatment with astaxanthin decreases protein levels of total and nuclear NF-κB in the hippocampus and cortex [136,141]. TNF-α [145], IL-1β [146], and IL-6 [147] are all pro-inflammatory cytokines produced by macrophages and are involved in the NF-κB pathway, and TNF-α and IL-1β have both been shown to activate NF-κB [144] and thus can operate in a positive feedback loop. In models of brain injury and disease, treatment with astaxanthin attenuated the increase in levels of TNF-α, IL-1β, and IL-6, exerting an anti-inflammatory effect [136,137,141]. Administration of astaxanthin also attenuates depressive-like behaviors in rodents by preventing neuroinflammation [148,149,150] and regulating survival pathways including PI3K/Akt [151,152]. Decrease in cytokine levels may be due to astaxanthin inhibiting invasion of macrophages, the source of cytokine production [136]. Persistent elevation of inflammatory cytokines has been shown to lead to demyelination and eventually cell death [153], and thus by lowering levels of pro-inflammatory cytokines astaxanthin protects against neuronal cell death. In addition, Zang et al. also suggest that astaxanthin prevents swelling of brain cells by downregulating the Na^+^-K^+^-2Cl^−^ co-transporter-1 (NKCC1), a key transporter implicated in brain edema, via the NF-κB pathway [141]. Inhibition of NKCC1 has been shown to attenuate disruption of the blood–brain barrier and thus provide a neuroprotective effect [154].

### 2.4. Lutein

Lutein is a plant-originated carotenoid found in green-leafy vegetables such as spinach, kale, broccoli, and other foods including eggs and avocados. Lutein contains an unsaturated hydrocarbon chain with cyclic structures at each end and is isomeric with zeaxanthin. Along with zeaxanthin, it is the only carotenoid that is found in the macula of the eye after crossing the blood–retina barrier [155]. Treatment with lutein attenuates retinopathy associated with photo stress, inflammation, and oxidative stress [156,157,158], and dietary lutein prevents aging- and diabetes-related eye diseases [159,160]. Lutein also crosses the blood–brain barrier to allow for delivery to various regions of the brain. Lutein is detected in the cortex, cerebellum, striatum, and hippocampus of rhesus monkeys after oral administration at 0.25–0.5 μmol/kg [161,162]. Therefore, studies investigating lutein’s benefits in brain development and potential protective effects during neurodegeneration are quickly becoming relevant [163,164].

Lutein decreases accumulation of lipid hydroperoxides and formation of malondialdehyde and thiobarbituric acid adduct, markers of lipid peroxidation, and it also inhibits oxygen photo-consumption in a concentration-dependent manner [165]. Lutein restores other antioxidants including glutathione, superoxide dismutase, and catalase in the brain during ischemic challenge [166]. Interestingly, Mohn et al. show that lutein levels in the mitochondrial membrane are inversely associated with fatty acid oxidation in the primate brain [162], indicating a potential protective role of lutein in brain pathology associated with mitochondrial dysfunction. Treatment with lutein significantly decreases mitochondrial ROS in *Caenorhabditis elegans (Caenorhabditis elegans)* lacking nuo-5/NDUFS1 and lpd-5/NDUFS4, genes that encode subunits of complex I in the electron transport chain [167]. This study demonstrates that depletion of complex I decreases mitochondrial ATP production, increases mitochondria ROS accumulation, and increases lipid peroxidation leading to synapse dysfunction, whereas treatment with lutein reverses these effects to rescue cholinergic synapse activity [167]. Lutein increases Nrf2 and decreases NF-κB subunit p65, thus, lutein may regulate transcription of genes that control antioxidant activity and inflammation [168,169,170,171,172].

Treatment with lutein protects neurons and brain-derived cells from apoptotic death. Lutein prevents loss of Bcl-2 and Bcl-xL, accumulation of Bax, and activation of caspases 3 and 8 in brain disease models of cerebral ischemia and Parkinson’s disease [172,173,174]. Caspase 3 is a central protease responsible for destruction of cellular structure and execution of apoptosis. In addition, caspase 3 disables functions of pro-survival proteins. Caspase 3 carries out post-translational cleavage of Bcl-xL and Bcl-2 into pro-death molecules [18,19,175,176,177]. Therefore, lutein-mediated caspase 3 inactivation may support the maintenance of full-length Bcl-xL and Bcl-2 and their anti-apoptotic properties in neurons. Furthermore, lutein may effectively reverse apoptosis triggered during glutamate-mediated excitotoxicity. During cerebral ischemia and neurodegeneration, excess glutamate activates N-methyl-d-aspartate (NMDA) receptors allowing a surge of intracellular calcium. This surge activates destructive enzymes including calpain and phospholipases to cause neuronal death. Lutein prevents loss of retinal ganglion cells against NMDA challenge [174] and suppresses death signaling including MAPK-mediated c-Jun activation [172,174]. In addition to apoptosis, Fung et al. showed that hypoxia-induced AMP activated protein kinase (AMPK) inhibits mTOR, followed by induction of microtubule-associated protein 1A/1B-light chain 3 (LC3) II, treatment with lutein protects glial cells by reversing this pathway [173]. LC3-I is the cytosolic form and LC3-II is the phosphatidylethanolamine conjugated form found in autophagosomes. Elevated ROS are shown to enhance recruitment of LC3 [178], therefore, both downregulation of LC3-II and attenuation of ROS in lutein treatment may prevent uncontrolled autophagic degradation of brain cells.

### 2.5. Fucoxanthin

Fucoxanthin is a carotenoid found in brown seaweed [179,180] with antioxidant and anti-inflammatory properties. Although functions of fucoxanthin are less researched compared to other carotenoids, an increasing number of studies suggest potential anti-apoptotic roles of fucoxanthin in neurodegenerative diseases [181,182,183,184]. Pharmacokinetic analysis of fucoxanthin and its metabolite fucoxanthinol in plasma after intragastric administration of fucoxanthin in rats has been performed [185,186], and tissue distribution of fucoxanthin after oral administration has also been performed using mouse models [187]. However, it is unknown if fucoxanthin crosses the blood–brain barrier. Despite limited information on fucoxanthin and the blood-brain barrier permeability, experiments have been performed that show that animals who undergo middle cerebral artery occlusion (MCAO) demonstrate brain tissue damage associated with apoptosis and neurological deficits, but fucoxanthin treatment reverses this MCAO-induced brain injury [188]. Treatment with fucoxanthin improves cognitive and motor function in animals challenged by neurodegeneration and ischemic insults [183,188,189]. Intragastric and intracerebroventricular administration of fucoxanthin reverses apoptosis in mouse traumatic brain injury and ischemic stroke models via enhancing the abundance of Bcl-2 and decreasing Bax, cytochrome c release, and caspase 3 activity [184,188]. Neuroblastoma SH-SY5Y cells treated with fucoxanthin show decreased numbers of pyknotic nuclei and condensation of chromatin, indicators of apoptosis, during ROS and amyloid-β challenge [181,182].

Fucoxanthin and its metabolites have strong radical scavenging activity [190]. For example, hydroxyl radical scavenging activity of fucoxanthin is 13.5 times greater than that of α-tocopherol [190]. In addition to scavenging ROS, fucoxanthin may directly occupy the docking site, altering protein function [191]. Fucoxanthin potentially binds directly to amyloid-β, preventing formation of pathological amyloid-β fibril [191]. Fucoxanthin also regulates intracellular redox status by regulating antioxidant-sensitive genes. Studies show that fucoxanthin activates transcription factor Nrf2 [184,188] and redox sensitive targets of Nrf2 such as heme oxygenase-1, superoxide dismutase, and Bcl-2 expression, protecting rodent brain against neurotoxic insult [184,188]. Fucoxanthin fails to reverse apoptosis in mice lacking Nrf2, indicating that Nrf2 is a critical part of fucoxanthin-mediated neuroprotection [184,188]. Fucoxanthin exhibits protective properties via activation of the PI3K/Akt pathway during neurotoxic stimulation [181,182]. PI3K/Akt promotes nuclear translocation of Nrf2 [192], thus, the PI3K/Akt pathway is a likely upstream component of fucoxanthin-mediated upregulation of antioxidant genes.

## 3. Conclusions

Neurons in the mature brain are generally postmitotic, and thus, maintaining viable neuron populations is critically important to support brain function and activity. During neurodegeneration, activation of apoptotic signaling causes structural and functional deterioration of the brain. To date, there is no treatment to cure neurodegenerative disease pathology. As an alternative to post-damage treatment, it is important to find ways to prevent neuronal apoptosis and alleviate apoptosis-induced cellular damage. In this review, we describe inhibitory roles of various carotenoids against neuronal apoptosis (Table 1). Carotenoids act as antioxidants to block upstream triggers of apoptosis and ROS-associated mitochondrial dysfunction. Carotenoids thus prevent energy deficit and subsequent activation of mitochondria-derived apoptotic molecules. In addition, carotenoids influence numerous transcription factors such as Nrf2, and NF-κB to regulate genes for apoptosis, antioxidant defense, and inflammation. Although we highlight roles of carotenoids in apoptotic signaling in this review, carotenoids may also intervene in other death pathways such as necroptosis, pyroptosis, and ferroptosis. Therefore, future investigation to distinguish the specific molecular mechanisms of neuronal death that are involved and continued translational studies in human subjects will be invaluable in outlining protective and therapeutic roles of carotenoids in neurodegeneration.

## Figures and Tables

**Table 1:** Current studies investigating roles of carotenoids in apoptotic pathways of brain related diseases.

	Preventing Loss of Bcl-2	Preventing Loss of Bcl-xL	Preventing Accumulation of Bax	Preventing Accumulation or Release of Cytochrome C	Preventing Accumulation or Activation of Caspase 3
β-carotene	[120]		[120]		[120]
Lycopene	[80,83,89]	[89]	[80,83,89]	[77,83,90]	[80,83]
Lutein	[172,173,174,193]	[173]	[173,174,193]	[174]	[173,193]
Astaxanthin	[140]		[136]		[137]
Zeaxanthin					[194]
Fucoxanthin	[188]		[188]	[184]	[184,188]
